# Supplementary figures and images for: Integrative bioinformatics and machine learning identify shared molecular mechanisms and diagnostic biomarkers between Helicobacter pylori infection and atrial fibrillation
Source: PLoS One. 2026 Apr 10;21(4):e0346038. doi: 10.1371/journal.pone.0346038 (PMC13068215; doi:10.1371/journal.pone.0346038)

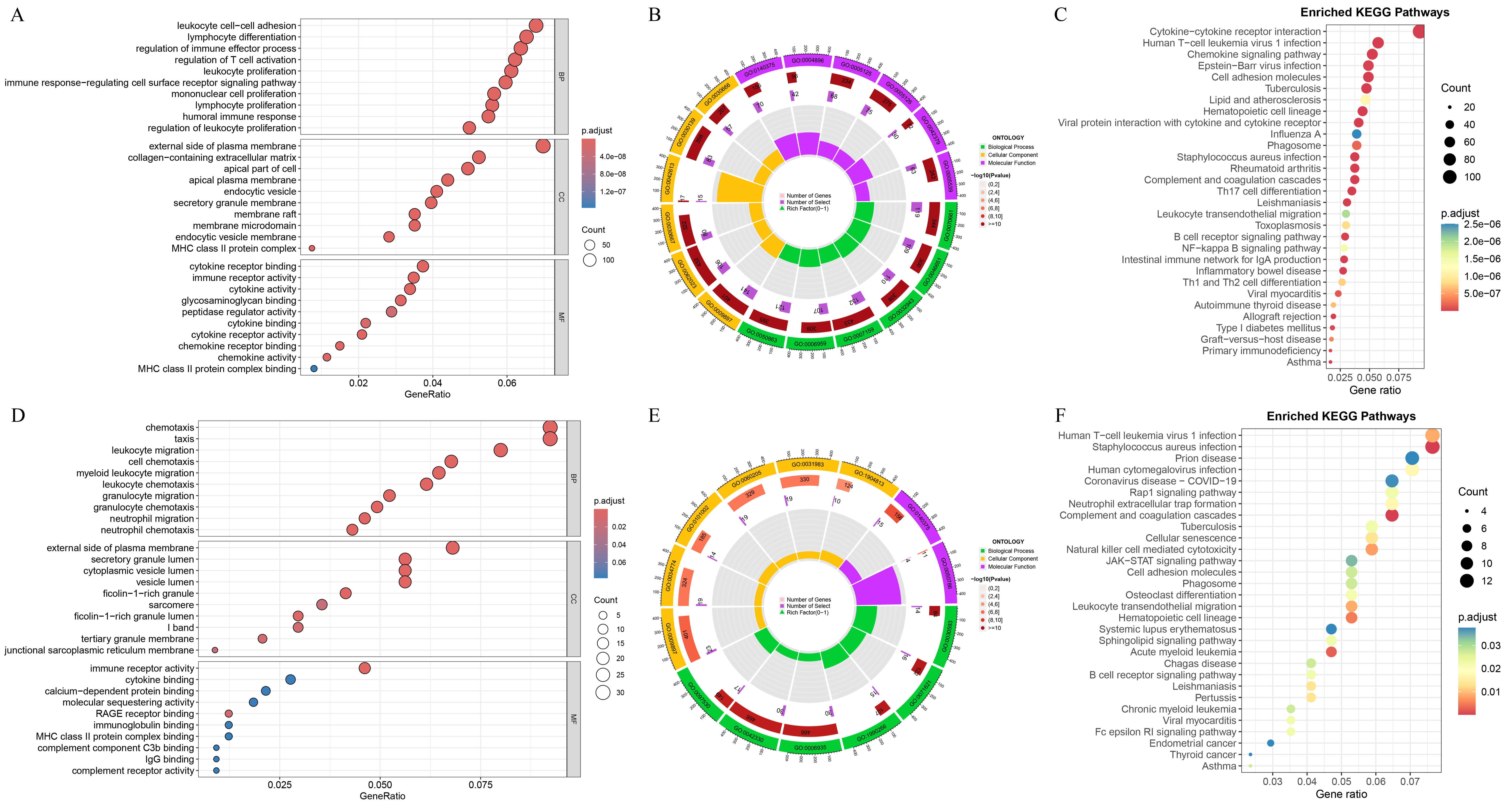

Supplement: S1 Fig — Dot plot (A, D) and circular plot (B, E) displaying the results of the GO enrichment analysis of DEGs specific to H. pylori infection and AF. KEGG enrichment analysis results of DEGs specific to H. pylori infection (C) and AF (F). (TIF) [file pone.0346038.s001.tif]

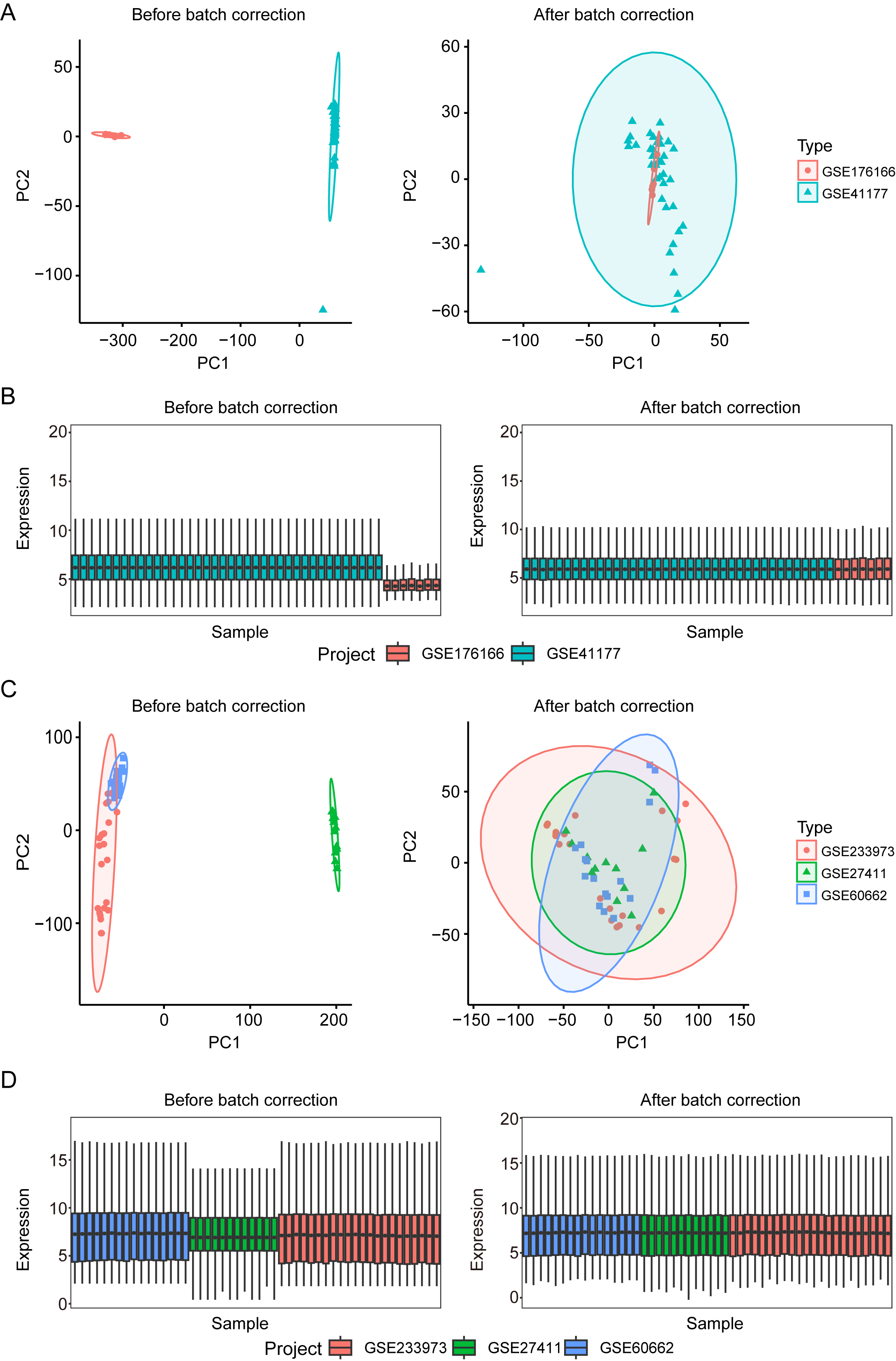

Supplement: S2 Fig — (TIF) [file pone.0346038.s002.tif]
