# Supplementary material for: Integrative bioinformatics and machine learning identify shared molecular mechanisms and diagnostic biomarkers between Helicobacter pylori infection and atrial fibrillation
Source: PLoS One. 2026 Apr 10;21(4):e0346038. doi: 10.1371/journal.pone.0346038 (PMC13068215; doi:10.1371/journal.pone.0346038)
Supplement: S1 Table — (DOCX) [file pone.0346038.s003.docx]

**Table S1. 77 union genes** **for comprehensive functional annotation and machine leaming.**

| **Gene name** | **Source** |
| --- | --- |
| SLA | DEGs |
| CSF2RA | DEGs |
| BAX | DEGs |
| ISOC2 | DEGs |
| FCER1G | DEGs |
| CLEC10A | DEGs |
| FCN1 | DEGs |
| S100A8 | DEGs |
| TYROBP | DEGs |
| C5AR1 | DEGs |
| PGAM1 | DEGs |
| BCL2A1 | DEGs |
| ALOX5AP | DEGs |
| S100A12 | DEGs |
| C1orf54 | DEGs |
| APBB1IP | DEGs |
| SERPINA1 | DEGs |
| C1QA | DEGs |
| S100A9 | DEGs |
| SELM | DEGs |
| C1QC | DEGs |
| ITGB2 | DEGs |
| HLA-DPB1 | DEGs |
| SELL | DEGs |
| PTPRCAP | DEGs |
| HCST | DEGs |
| G6PD | DEGs |
| AOAH | DEGs |
| VNN2 | DEGs |
| ID1 | DEGs |
| IL17F | DEGs |
| C1QB | DEGs |
| MS4A4A | DEGs |
| CD74 | DEGs & WGCNA |
| NCF2 | DEGs |
| ITGAM | DEGs |
| PYCARD | DEGs |
| CDKN1A | DEGs |
| HCK | DEGs |
| PROK2 | DEGs |
| VSIG4 | DEGs |
| MS4A7 | DEGs |
| CD28 | DEGs |
| SPI1 | DEGs |
| FCGR3A | DEGs |
| GPR171 | DEGs |
| CLC | DEGs |
| CD3D | DEGs |
| MNDA | DEGs |
| CLEC4A | DEGs |
| MGAM | DEGs |
| LAPTM5 | DEGs |
| FMO3 | DEGs |
| IL7R | DEGs |
| CSF3R | DEGs |
| HLA-DPA1 | DEGs |
| CMTM2 | DEGs |
| CSF2RB | DEGs |
| CYP4F3 | DEGs |
| RAC2 | DEGs |
| RGS18 | DEGs |
| SAMSN1 | DEGs |
| CD2 | DEGs |
| TREM1 | DEGs |
| TAAR2 | DEGs |
| XK | DEGs |
| MCOLN3 | DEGs |
| DNER | DEGs |
| ENPP5 | DEGs |
| SLC26A9 | DEGs |
| GABARAPL1 | DEGs |
| TCEAL2 | DEGs |
| RYR2 | DEGs |
| SOD2 | WGCNA |
| CHL1 | WGCNA |
| RLN2 | WGCNA |
| IGLL1 | WGCNA |
