# Supplementary material for: Integrative bioinformatics and machine learning identify shared molecular mechanisms and diagnostic biomarkers between Helicobacter pylori infection and atrial fibrillation
Source: PLoS One. 2026 Apr 10;21(4):e0346038. doi: 10.1371/journal.pone.0346038 (PMC13068215; doi:10.1371/journal.pone.0346038)
Supplement: S2 Table — (DOCX) [file pone.0346038.s004.docx]

**S2 Table. AUC values for various models across cohorts in H. pylori infection.**

| **Model** | **AUC in Training Cohort** | **AUC in GSE60427** | **AUC in GSE5081** | **Average AUC** |
| --- | --- | --- | --- | --- |
| RF+NaiveBayes | 0.985 | 0.816 | 0.995 | 0.932 |
| RF+plsRglm | 0.985 | 0.797 | 0.995 | 0.925 |
| Ridge | 0.980 | 0.793 | 1.000 | 0.924 |
| Enet[alpha=0.1] | 1.000 | 0.777 | 0.979 | 0.919 |
| RF | 1.000 | 0.758 | 0.990 | 0.916 |
| plsRglm | 0.995 | 0.762 | 0.990 | 0.915 |
| Enet[alpha=0.7] | 1.000 | 0.754 | 0.984 | 0.913 |
| NaiveBayes | 0.958 | 0.777 | 1.000 | 0.912 |
| Enet[alpha=0.6] | 1.000 | 0.750 | 0.984 | 0.911 |
| Enet[alpha=0.4] | 1.000 | 0.750 | 0.979 | 0.910 |
| Enet[alpha=0.3] | 1.000 | 0.754 | 0.974 | 0.909 |
| Enet[alpha=0.8] | 1.000 | 0.742 | 0.984 | 0.909 |
| Enet[alpha=0.2] | 1.000 | 0.750 | 0.974 | 0.908 |
| LDA | 1.000 | 0.768 | 0.948 | 0.905 |
| RF+Ridge | 0.997 | 0.727 | 0.984 | 0.903 |
| Enet[alpha=0.5] | 1.000 | 0.723 | 0.984 | 0.902 |
| Enet[alpha=0.9] | 1.000 | 0.723 | 0.984 | 0.902 |
| RF+Enet[alpha=0.2] | 1.000 | 0.715 | 0.984 | 0.900 |
| RF+Enet[alpha=0.6] | 1.000 | 0.715 | 0.984 | 0.900 |
| RF+Enet[alpha=0.7] | 1.000 | 0.715 | 0.984 | 0.900 |
| RF+glmBoost | 1.000 | 0.715 | 0.984 | 0.900 |
| RF+Enet[alpha=0.8] | 1.000 | 0.715 | 0.984 | 0.900 |
| Lasso+NaiveBayes | 1.000 | 0.719 | 0.979 | 0.899 |
| RF+Enet[alpha=0.1] | 0.998 | 0.715 | 0.984 | 0.899 |
| RF+Enet[alpha=0.3] | 1.000 | 0.711 | 0.984 | 0.898 |
| RF+Enet[alpha=0.5] | 1.000 | 0.711 | 0.984 | 0.898 |
| RF+Enet[alpha=0.4] | 1.000 | 0.711 | 0.984 | 0.898 |
| glmBoost | 1.000 | 0.715 | 0.979 | 0.898 |
| GBM | 1.000 | 0.703 | 0.984 | 0.896 |
| Lasso | 1.000 | 0.695 | 0.990 | 0.895 |
| RF+GBM | 1.000 | 0.699 | 0.984 | 0.895 |
| RF+Enet[alpha=0.9] | 1.000 | 0.691 | 0.984 | 0.892 |
| Lasso+glmBoost | 1.000 | 0.684 | 0.984 | 0.889 |
| Lasso+LDA | 1.000 | 0.699 | 0.969 | 0.889 |
| Lasso+GBM | 1.000 | 0.703 | 0.953 | 0.885 |
| Lasso+Stepglm[forward] | 1.000 | 0.684 | 0.971 | 0.885 |
| XGBoost | 1.000 | 0.662 | 0.987 | 0.883 |
| RF+LDA | 1.000 | 0.727 | 0.922 | 0.883 |
| SVM | 0.984 | 0.656 | 0.958 | 0.866 |
| RF+Stepglm[forward] | 1.000 | 0.639 | 0.958 | 0.866 |
| Lasso+XGBoost | 1.000 | 0.637 | 0.927 | 0.855 |
| Lasso+plsRglm | 1.000 | 0.582 | 0.938 | 0.840 |
| Lasso+SVM | 1.000 | 0.562 | 0.938 | 0.833 |
| RF+SVM | 1.000 | 0.562 | 0.917 | 0.826 |
| RF+XGBoost | 0.958 | 0.625 | 0.792 | 0.791 |
| Stepglm[forward] | 1.000 | 0.646 | 0.625 | 0.757 |
