# Supplementary material for: Integrative bioinformatics and machine learning identify shared molecular mechanisms and diagnostic biomarkers between Helicobacter pylori infection and atrial fibrillation
Source: PLoS One. 2026 Apr 10;21(4):e0346038. doi: 10.1371/journal.pone.0346038 (PMC13068215; doi:10.1371/journal.pone.0346038)
Supplement: S3 Table — (DOCX) [file pone.0346038.s005.docx]

**S3 Table. AUC values for various models across cohorts in AF.**

| **Model** | **AUC in Training Cohort** | **AUC in GSE60427** | **AUC in GSE5081** | **Average AUC** |
| --- | --- | --- | --- | --- |
| RF+LDA | 1.000 | 0.840 | 0.800 | 0.880 |
| glmBoost+Enet[alpha=0.9] | 0.991 | 0.800 | 0.800 | 0.864 |
| glmBoost+Lasso | 0.991 | 0.800 | 0.800 | 0.864 |
| glmBoost+Enet[alpha=0.8] | 0.989 | 0.840 | 0.760 | 0.863 |
| glmBoost+Enet[alpha=0.7] | 0.989 | 0.800 | 0.760 | 0.850 |
| RF+Stepglm[forward] | 1.000 | 0.780 | 0.740 | 0.840 |
| Enet[alpha=0.3] | 0.991 | 0.880 | 0.640 | 0.837 |
| Enet[alpha=0.4] | 0.991 | 0.880 | 0.640 | 0.837 |
| glmBoost+Enet[alpha=0.5] | 0.991 | 0.800 | 0.720 | 0.837 |
| glmBoost+Enet[alpha=0.6] | 0.991 | 0.800 | 0.720 | 0.837 |
| RF+Enet[alpha=0.8] | 0.991 | 0.760 | 0.760 | 0.837 |
| RF+Enet[alpha=0.2] | 0.989 | 0.800 | 0.720 | 0.836 |
| RF+Enet[alpha=0.3] | 0.989 | 0.800 | 0.720 | 0.836 |
| RF+Enet[alpha=0.4] | 0.989 | 0.800 | 0.720 | 0.836 |
| plsRglm | 1.000 | 0.900 | 0.580 | 0.827 |
| RF | 1.000 | 0.800 | 0.680 | 0.827 |
| Enet[alpha=0.1] | 0.991 | 0.880 | 0.600 | 0.824 |
| Enet[alpha=0.2] | 0.991 | 0.880 | 0.600 | 0.824 |
| Enet[alpha=0.5] | 0.989 | 0.840 | 0.640 | 0.823 |
| RF+Enet[alpha=0.6] | 0.989 | 0.760 | 0.720 | 0.823 |
| RF+Enet[alpha=0.7] | 0.989 | 0.760 | 0.720 | 0.823 |
| RF+Enet[alpha=0.5] | 0.989 | 0.760 | 0.720 | 0.823 |
| Lasso | 0.977 | 0.760 | 0.720 | 0.819 |
| glmBoost+Ridge | 0.994 | 0.800 | 0.640 | 0.811 |
| glmBoost+Enet[alpha=0.1] | 0.994 | 0.800 | 0.640 | 0.811 |
| glmBoost+Enet[alpha=0.3] | 0.991 | 0.800 | 0.640 | 0.810 |
| glmBoost+Enet[alpha=0.2] | 0.991 | 0.800 | 0.640 | 0.810 |
| glmBoost+Enet[alpha=0.4] | 0.991 | 0.800 | 0.640 | 0.810 |
| RF+Enet[alpha=0.1] | 0.991 | 0.800 | 0.640 | 0.810 |
| Enet[alpha=0.6] | 0.989 | 0.800 | 0.640 | 0.810 |
| Enet[alpha=0.9] | 0.983 | 0.760 | 0.680 | 0.808 |
| RF+Enet[alpha=0.9] | 0.983 | 0.760 | 0.680 | 0.808 |
| RF+Lasso | 0.974 | 0.800 | 0.640 | 0.805 |
| RF+Stepglm[both] | 1.000 | 0.700 | 0.700 | 0.800 |
| RF+Stepglm[backward] | 1.000 | 0.700 | 0.700 | 0.800 |
| Lasso+glmBoost | 0.991 | 0.760 | 0.640 | 0.797 |
| glmBoost | 0.991 | 0.760 | 0.640 | 0.797 |
| Lasso+plsRglm | 0.991 | 0.880 | 0.520 | 0.797 |
| Enet[alpha=0.8] | 0.989 | 0.760 | 0.640 | 0.796 |
| Enet[alpha=0.7] | 0.989 | 0.760 | 0.640 | 0.796 |
| glmBoost+LDA | 0.986 | 0.760 | 0.640 | 0.795 |
| Ridge | 0.949 | 0.840 | 0.560 | 0.783 |
| RF+glmBoost | 0.989 | 0.720 | 0.640 | 0.783 |
| NaiveBayes | 0.966 | 0.740 | 0.640 | 0.782 |
| Lasso+LDA | 0.989 | 0.720 | 0.600 | 0.770 |
| RF+Ridge | 0.986 | 0.800 | 0.520 | 0.769 |
| RF+NaiveBayes | 0.977 | 0.800 | 0.520 | 0.766 |
| Lasso+NaiveBayes | 0.991 | 0.680 | 0.600 | 0.757 |
| XGBoost | 0.937 | 0.700 | 0.620 | 0.752 |
| Lasso+SVM | 0.950 | 0.700 | 0.600 | 0.750 |
| RF+plsRglm | 0.966 | 0.760 | 0.520 | 0.749 |
| glmBoost+plsRglm | 0.991 | 0.720 | 0.520 | 0.744 |
| Lasso+Stepglm[forward] | 1.000 | 0.800 | 0.420 | 0.740 |
| Stepglm[forward] | 1.000 | 0.700 | 0.500 | 0.733 |
| RF+XGBoost | 0.941 | 0.580 | 0.620 | 0.714 |
| glmBoost+Stepglm[forward] | 1.000 | 0.700 | 0.420 | 0.707 |
| glmBoost+NaiveBayes | 0.991 | 0.560 | 0.560 | 0.704 |
| RF+SVM | 0.900 | 0.600 | 0.600 | 0.700 |
| Lasso+XGBoost | 0.941 | 0.500 | 0.620 | 0.687 |
| glmBoost+XGBoost | 0.941 | 0.500 | 0.620 | 0.687 |
| SVM | 0.850 | 0.600 | 0.600 | 0.683 |
| glmBoost+SVM | 0.950 | 0.500 | 0.600 | 0.683 |
| LDA | 1.000 | 0.560 | 0.460 | 0.673 |
