# Supplementary material for: Integrative bioinformatics and machine learning identify shared molecular mechanisms and diagnostic biomarkers between Helicobacter pylori infection and atrial fibrillation
Source: PLoS One. 2026 Apr 10;21(4):e0346038. doi: 10.1371/journal.pone.0346038 (PMC13068215; doi:10.1371/journal.pone.0346038)
Supplement: S4 Table — (DOCX) [file pone.0346038.s006.docx]

**S4 Table. The AUC values of the top 5 genes incorporated in the optimal ML methods for H. pylori infection.**

| **Gene Name** | **AUC value** |
| --- | --- |
| S100A9 | 0.913 |
| S100A8 | 0.907 |
| C1QA | 0.944 |
| HLA−DPA1 | 0.971 |
| CD74 | 0.915 |
