# Supplementary material for: Integrative bioinformatics and machine learning identify shared molecular mechanisms and diagnostic biomarkers between Helicobacter pylori infection and atrial fibrillation
Source: PLoS One. 2026 Apr 10;21(4):e0346038. doi: 10.1371/journal.pone.0346038 (PMC13068215; doi:10.1371/journal.pone.0346038)
Supplement: S5 Table — (DOCX) [file pone.0346038.s007.docx]

**S5 Table. The AUC values of the top 5 genes incorporated in the optimal ML methods for AF.**

| **Gene Name** | **AUC value** |
| --- | --- |
| PGAM1 | 0.857 |
| S100A8 | 0.906 |
| SLA | 0.917 |
| C5AR1 | 0.886 |
| CD28 | 0.849 |
