# Supplementary material for: Integrative bioinformatics and machine learning identify shared molecular mechanisms and diagnostic biomarkers between Helicobacter pylori infection and atrial fibrillation
Source: PLoS One. 2026 Apr 10;21(4):e0346038. doi: 10.1371/journal.pone.0346038 (PMC13068215; doi:10.1371/journal.pone.0346038)
Supplement: Attachment — Submitted filename: To the Reviewers.docx [file pone.0346038.s009.docx]

**To the Reviewers：**

We sincerely thank the reviewer for the time and effort invested in reviewing our manuscript and for providing such thorough and constructive feedback. We fully acknowledge the serious concerns raised regarding the presentation, structure, and overall readiness of our initial submission. These comments have been invaluable in guiding a fundamental revision of our work.

In direct response to your critique that the manuscript did not meet minimum journal standards, we undertook a comprehensive overhaul. We have systematically addressed every point raised by carefully re-examining, re-writing, and professionally refining the entire manuscript. Below, we provide a detailed point-by-point account of the actions taken.

**Response to Reviewer #1:**

**Comment 1:** On overall structure, language, grammar, and editorial quality.
Response: We agree completely with the reviewer's assessment that the original manuscript's language and structure were substandard and read more like a project report. To rectify this, we have not merely edited but have substantially re-written the manuscript to conform to the standards of a research article.

**Global Language and Formatting Revision:** The entire text has undergone rigorous professional language editing and proofreading. We have corrected grammatical errors, improved sentence fluency and clarity, eliminated awkward phrasing and repetition, and ensured consistent and formal scientific terminology throughout.

**Restructured Methods Section:** The "Materials and Methods" section has been completely re-organized and re-written. It now provides a coherent, logical narrative of our analytical workflow in dedicated subsections, moving decisively away from the initial checklist format to offer clear, reproducible descriptions.

**Re-organized and Re-written Results Section:** We have introduced clear, descriptive subheadings that reflect the analytical progression The text has been carefully re-crafted to ensure a logical flow, explicitly connecting each analytical step to the next and guiding the reader clearly through the sequence of discoveries.

**Expanded and Deepened Discussion Section:** We have significantly expanded the "Discussion" to move decisively beyond re-stating results. It now provides a critical interpretation of our findings within the broader context of existing literature, elaborates in depth on the biological and clinical implications (e.g., the dual contextual role of S100A8), and clearly differentiates between evidence-based conclusions and speculative points for future inquiry. A dedicated "Limitations and Future Perspectives" subsection has been added to frame the study appropriately.

**Correction of Editorial Artifacts:** Regarding the specific typographical error ("Fiugre") and improper figure references noted, we have meticulously proofread our source documents. We believe these may have been introduced during the initial file export or submission process. We will exercise utmost care during re-submission to ensure all text, labels, and citations are error-free.

**Comment 2:** On the presentation of figures/tables and the overall logical/visual flow.
Response: We thank the reviewer for this critical feedback on presentation, which we have addressed comprehensively.

**Enhanced Figures and Tables:** All figures have been re-created and formatted strictly according to typical journal requirements concerning font consistency, size, resolution, and visual clarity. The legends have been entirely re-written to be self-contained and explanatory, detailing what each panel shows and summarizing the key scientific finding it conveys.

**Improved Logical Narrative and Cohesion:** To address the disjointed flow, we have strengthened the connective tissue of the manuscript. This includes refining the narrative arc from the Introduction through to the Discussion and incorporating more transitional phrasing between sections and paragraphs. This ensures a smoother, more coherent reading experience that logically connects the biological question, the computational strategy, and the final interpretation.

We are profoundly grateful for the reviewer's rigorous assessment. The critical feedback provided was essential for us to elevate the quality of our work. We believe the extensive revisions detailed above have thoroughly addressed all the concerns raised and have transformed the manuscript into a polished, coherent, and publication-ready research article. We hope the revised version now meets the expected standards of your journal.

**Response to Reviewer #2:**

We sincerely thank the reviewer for the positive assessment and exceptionally constructive suggestions, which have been instrumental in guiding our comprehensive revision. As noted in our response to Reviewer #1, we have re-written the manuscript in its entirety to address overarching presentation issues. Your specific comments were central to shaping the new, detailed methodological reporting within this re-written framework, greatly enhancing the work's transparency and reproducibility.

**Comment 1:** Strengthening transparency in dataset description and preprocessing.
Response: We have added a new subsection, and a Supplementary Table 1 summarizing each dataset (accession, platform, sample sizes). We now clearly state that ComBat batch correction was applied to the discovery cohorts only, while validation cohorts were processed independently.

**Comment 2:** Clarifying the machine learning workflow.
Response: We have expanded the methods subsection “Machine Learning-Based Diagnostic Model Construction and Validation” to detail the process:

Hyperparameter tuning was performed using 10-fold cross-validation repeated 5 times within the discovery cohort.

Final gene panels were derived by intersecting top features from multiple algorithms.

The integrated models were implemented via a two-step approach (base RF + meta-classifier).

All reported AUROC values are from the independent validation cohorts, ensuring an unbiased performance estimate.

**Comment 3:** Improving language and formatting consistency.
Response: The manuscript has undergone thorough proofreading. All typographical errors have been corrected, and formatting (abbreviations, spacing, figure labels) has been standardized throughout.

We are grateful for your insightful comments, which have strengthened the manuscript.
