# Supplementary material for: Integrative bioinformatics and machine learning identify shared molecular mechanisms and diagnostic biomarkers between Helicobacter pylori infection and atrial fibrillation
Source: PLoS One. 2026 Apr 10;21(4):e0346038. doi: 10.1371/journal.pone.0346038 (PMC13068215; doi:10.1371/journal.pone.0346038)
Supplement: Attachment — Submitted filename: To_the_Reviewers_auresp_2.docx [file pone.0346038.s010.docx]

**Response to Reviewer #1：**

Dear Reviewer,

We sincerely thank you for re-reviewing our manuscript and for providing such valuable feedback. Your professional insights have been instrumental in further improving the quality of our work. In this second round of revision, we have carefully considered all the issues raised and have made corresponding improvements to the manuscript. Below is our point-by-point response to your specific comments.

**Response to Reviewer #1:**

**Comment 1:** The manuscript lacks clear, publication-ready data and tables to adequately support the results. Key findings are not summarized in structured tables or interpretable figures, which limits the evidentiary value of the manuscript.

**Response:** We fully agree with your assessment that clear data visualization and structured tables are fundamental to supporting scientific conclusions. To address this, we have comprehensively supplemented and optimized the figures and tables in our manuscript: **Addition of a study flowchart (now Figure 1)**: We have included a comprehensive flowchart that systematically illustrates the entire research workflow, from data acquisition and preprocessing, through differential expression analysis, WGCNA, machine learning, and hub gene screening, to drug prediction. This visual summary helps readers quickly grasp the overall study design. **Tabular presentation of machine learning features and performance (new S2 Table and S3 Table):** The pivotal diagnostic genes for H. pylori and AF (previously shown only in Figure 4A and 4F) are now summarized, which also lists each gene's SHAP value and ranking, enabling quantitative assessment of feature importance. The diagnostic performance metrics (AUROC) of the final models are now organized in S4 Table and S5 Table, with results clearly labeled for both the discovery cohort (internal cross-validation) and independent validation cohorts. This makes the model validation process more transparent and credible. **Visualization of batch effect correction (new Fig S2):** To address concerns regarding dataset heterogeneity, we have added PCA plots and boxplots for both AF and H. pylori datasets before and after ComBat correction. This figure intuitively demonstrates that batch effects were effectively eliminated and samples from different datasets were well-integrated, providing visual justification for subsequent combined analyses. **Complete list of core genes (new S1 Table):** We have provided the full list of 77 union genes (including gene symbols, full names, and expression patterns) as a supplementary table, facilitating direct access and analysis by readers. All figures and tables have been prepared according to journal formatting guidelines (appropriate resolution, font size, layout), and the legends have been rewritten to be self-explanatory. We believe these additions substantially enhance the readability and evidentiary value of our results.

**Comment 2:** The Discussion remains largely narrative. It would benefit from deeper analysis rather than speculative expansion. In particular, the Limitations section should be substantially expanded to address dataset heterogeneity, model stability, lack of causal inference, and the non-predictive nature of molecular docking.

**Response:** We thank you for your insightful comments on the Discussion. In our revision, we have placed particular emphasis on enhancing the depth and critical analysis of this section: **Strengthened interpretation grounded in results:** The newly added figures and tables (Figure 1 flowchart, Tables 2 and 3, Supplementary Figure 2, etc.) provide a more robust data foundation for the core arguments in the Discussion. We have closely integrated these new results into the Discussion, offering in-depth interpretations of key findings (such as the dual role of S100A8 and the immune network status of hub genes) while avoiding unsubstantiated speculation. **Clear distinction between conclusions and hypotheses:** We have carefully reviewed all discussion statements to ensure that every inference is grounded in our results or published literature. Hypothetical points (such as the therapeutic potential of candidate drugs) are expressed with appropriate caution, explicitly noting the need for future validation. **Expanded limitations discussion:** Following your suggestion, we have further emphasized the following aspects in the "Limitations and Future Perspectives" subsection: Dataset heterogeneity: Although ComBat correction was applied, the original data originated from different platforms; future validation using prospective, uniformly processed cohorts is still warranted. Model stability: While the models performed well in independent validation, broader external validation in multi-center studies would further strengthen their generalizability. Lack of causal inference: This study is associational and cannot establish causality; future studies employing Mendelian randomization or longitudinal designs are needed to explore causal relationships. Predictive nature of molecular docking: The docking results are computational predictions; their biological activity, specificity, and safety require experimental validation.

These additions have made the Limitations section more comprehensive and balanced, reflecting our clear awareness of the scientific boundaries of our conclusions.

We once again thank you for taking the time to review our manuscript. Your rigorous and detailed feedback has pushed us to pursue higher standards. We would also like to express our sincere gratitude to **Reviewer #2** for their positive evaluation and recommendation for publication. The professional insights from both reviewers have jointly contributed to the improvement of this study, and we are deeply appreciative.

We hope that the revised manuscript now meets your expectations.

Sincerely.
